# Exploring Gut Microbiota-Targeted Therapies for Canine Idiopathic Epilepsy

**DOI:** 10.3390/ijms26041742

**Published:** 2025-02-18

**Authors:** Luann Blanquet, Diana Serra, Carla Marrinhas, Anabela Almeida

**Affiliations:** 1 EUVG—Escola Universitária Vasco de Gama, Campus Universitário, Av. José R. Sousa Fernandes 197, 3020-210 Coimbra, Portugal; 2 CIVG—Centro de Investigação Vasco da Gama, EUVG—Escola Universitária Vasco da Gama, Campus Universitário-Bloco B, Av. José R. Sousa Fernandes 197, 3020-210 Coimbra, Portugal; 3 CNC—Centro de Neurociências e Biologia Celular, Universidade de Coimbra, 3004-531 Coimbra, Portugal; 4 Onevet Hospital Veterinário do Baixo Vouga, Estrada Nacional 1, 355, 3750-742 Águeda, Portugal; 5 CIBIT/ICNAS—Instituto de Imagem Biomédica e Investigação Translacional de Coimbra, Universidade de Coimbra, Polo 3, Azinhaga de Santa Comba, 3000-548 Coimbra, Portugal

**Keywords:** dogs, epilepsy, microbiota–gut–brain axis, gut microbiota, gut-related therapeutic strategies, ketogenic diet, fecal microbiota transplant, probiotics

## Abstract

Epilepsy stands out as one of the most prevalent chronic neurological conditions affecting companion animals. Recent research has increasingly focused on exploring the role of gut microbiota in influencing neurological conditions, like epilepsy. This influence stems from the bidirectional communication pathways between gut bacteria and the brain, which involve metabolic, neural, immunological, and endocrine mechanisms. In fact, a balanced and stable gut microbiota is essential to maintaining normal gut physiology and ensuring appropriate signaling along the gut–brain axis. Conversely, dysbiosis can have detrimental effects on gut physiology and may contribute to the development or exacerbation of neurological conditions, including epilepsy. Considering these findings, this review article aims to deepen the understanding of the mechanisms underlying the microbiota–gut–brain connection in the context of canine idiopathic epilepsy. Moreover, this review presents recent data on innovative gut-related therapeutic strategies for canine idiopathic epilepsy treatment.

## 1. Introduction

Canine epilepsy is a complex and challenging neurological disorder that affects approximately 0.6–0.75% of the dog population [1]. Characterized by recurrent seizures, this condition can have a great impact on the well-being and quality of life of both the affected animals and their caregivers. Although conventional treatments, such as those using antiepileptic drugs, have been implemented to manage seizure activity, they often have limitations, achieving incomplete seizure control and leading to adverse effects, including sedation, ataxia, and hepatotoxicity [2]. Therefore, there is an urgent need for a deeper understanding of the underlying mechanisms of epilepsy in dogs and for novel therapeutic approaches that offer improved efficacy and fewer side effects.

In recent years, research into the role of gut microbiota in health and disease has gained considerable momentum across various fields of medicine, including veterinary science [3]. The gut microbiota, a complex ecosystem of microorganisms inhabiting the gastrointestinal tract, is recognized for its essential contributions to digestion, metabolism, immune function, and overall host well-being [3]. Moreover, emerging evidence suggests a potential link between alterations in gut microbiota composition and the pathogenesis of neurological disorders, including epilepsy, particularly in mice and in humans [4]. In the context of canine epilepsy, researchers have begun to explore how gut microbiota dysregulation may influence seizure susceptibility, frequency, and severity in affected dogs [5]. This evidence paves the way for the identification of novel therapeutic targets and strategies to improve clinical outcomes.

This review aims to provide a comprehensive overview of the current understanding of the relationship between canine idiopathic epilepsy and gut microbiota modification. By synthesizing findings from recent research studies and clinical observations, we seek to elucidate the potential mechanisms by which alterations in the gut microbiota may contribute to the pathogenesis of epilepsy in dogs. Furthermore, we will explore the implications of these findings for the diagnosis, treatment, and management of canine epilepsy, with a focus on identifying opportunities for future research and clinical practice in veterinary medicine. Through this study, it will be possible to increase the understanding of the complex interactions between the gut microbiota and neurological health in dogs, ultimately improving medical care for dogs with idiopathic epilepsy. The following keywords were used to establish the references’ list with the use of the PubMed, PMC, and Web of Science search engines: “gut microbiota”, “canine”, “dog”, “epilepsy”, “probiotics”, “fecal microbiota transplant”, and “ketogenic diet”.

## 2. Basics of the Gut Microbiota

### 2.1. Canine Gut Microbiota

The canine gut microbiota is a vital component of a complex ecosystem that includes intestinal epithelial cells, the mucus layer, the immune system, and the luminal environment. This is influenced by numerous factors, namely, the diet; drugs, such as antibiotics; and acute episodes of diarrhea [3,6,7]. The gastrointestinal tract of dogs hosts a diverse community of microorganisms, including bacteria, virus, fungi, and protozoa. Bacteria are the predominant microorganisms, accounting for over 98% of metagenomic sequencing reads from fecal samples in dogs [3,7].

The gut microbiota is primarily composed of strict or facultative anaerobic bacteria, particularly in the large intestine. Differences in bacterial populations exist between the stomach and the small and large intestines due to variations in intestinal physiology, such as oxygen levels, pH, antimicrobial compounds, and motility [8].

Despite advances in molecular techniques, a full characterization of the diverse bacterial populations in the intestinal microbiota remains challenging. Many studies on canine intestinal microbiota employ the 16S rRNA gene comparison technique, which has a limited resolution to identify bacteria at the species level [8].

Phylogenetic analyses have identified predominant bacterial phyla in healthy canine intestines, including *Firmicutes*, *Fusobacteria*, and *Bacteroidetes* [6]. These phyla vary across different regions of the intestine, with *Firmicutes* dominating the duodenum and jejunum, and *Fusobacteria* and *Bacteroidetes* being more prevalent in the ileum and colon [9].

Fungal populations represent approximately 2% of canine gut microbiota, primarily consisting of *Ascomycota*. Other fungal phyla, such as *Glomeromycota* and *Zygomycota* have also been identified in dogs, with genera like *Nacaseomyces* and *Candida* being predominant [10].

Despite the growing understanding of the canine gut microbiota, there is still limited knowledge about its development and acquisition. Most studies carried out focusing on puppies reveal significant inter-individual variability in early fecal microbiota, with diversity and stability increasing with age [3].

In addition, research by Swanson et al. [9] showed that the gastrointestinal metagenomes present high similarity between humans and dogs. Given the similarities between human and dog canine gut microbiota, the knowledge gained from decoding the canine gut microbiota can be important from a translational point of view, i.e., to better understand the human gut microbiota composition and its modification caused by various factors, such as the diet, and for the development of gut microbiota-targeted effective therapies to improve canine and human health and well-being.

### 2.2. Essential Functions of the Canine Gut Microbiota

The canine gut microbiota is a complex and dynamic community of microorganisms that plays a pivotal role in maintaining overall health and well-being. One of its essential functions is aiding digestion through hosting metabolism pathways that enable the breakdown of complex carbohydrates and other nutrients which the host cannot digest on its own, thereby producing energy and beneficial substrates for both bacterial proliferation and the host [6]. Furthermore, the gut microbiota synthesizes essential vitamins, including B complex vitamins, which are crucial to various physiological processes in the host [11]. Additionally, it transforms primary bile acids, such as cholic acid and chenodeoxycholic acid, into secondary bile acids that are critical to the absorption of dietary fats and fat-soluble vitamins in the gut [12].

The gut microbiota also protects the host against pathogenic bacteria by producing bacteriocins and colicins and competing for nutrients, thus providing ecological benefits [13]. It also influences the induction, shaping, and function of the host’s immune system, playing a vital role in distinguishing between pathogens and commensal bacteria and contributing to the development of robust gut barrier and immune response [10,14]. Physiological impairments related to the gut barrier and gut immune response have been related to the onset of diseases, such as the case of chronic inflammatory enteropathies in dogs [15].

In addition, the gut microbiota produces short-chain fatty acids (SCFAs), such as acetate, propionate, and butyrate, through the fermentation of alcohols and non-digestible carbohydrates, which play a crucial role in gut homeostasis [8]. In fact, SCFAs provide energy to the intestinal epithelium and other tissues, support the growth and differentiation of intestinal cells, and contribute to constant intestinal pH. They also serve as a sensory defense mechanism and facilitate the re-absorption of Na+ or K+ ions through the intestine, contributing to electrolyte balance and fluid homeostasis. Furthermore, these fatty acids have anti-inflammatory properties that help to maintain good intestinal health. The anti-inflammatory properties of SCFAs stem from their ability to regulate immune responses, enhance intestinal barrier integrity, reduce oxidative stress, and modulate metabolite production [16].

In summary, the canine gut microbiota plays a vital role in several processes, including digestion, nutrient absorption, metabolism, immune system regulation, and defense against pathogens, highlighting its high relevance as a metabolically active “organ” essential to canine health.

### 2.3. Communication Between the Gut and the Brain via the Microbiota–Gut–Brain Axis in Dogs

Communication between the brain and the gut is a complex and bidirectional process involving several components, including the central nervous system, the enteric nervous system, the endocrine system, and the immune system. These systems communicate through neuronal, hormonal, and immunological signals [17].

Recent findings suggest that the gut microbiota may have a high impact on the gut–brain axis, and for this reason, recent research has designated this axis as the “microbiota–gut–brain axis”. In fact, the gut microbiota, composed of trillions of microorganisms, can produce metabolites that may impact brain function, putatively regulating many aspects of health, including behavior/aggressivity [18] and stress response [19] in dogs and in mice, respectively.

A recent study by Mondo et al. [18] found a correlation between dysbiosis (imbalance in bacterial composition) and aggressive or phobic behavior in dogs. Their study identified abnormal bacterial structures, high variability, and increased levels of certain bacteria in the fecal samples of aggressive dogs, particularly *Catenibacterium* and *Megamonas*. In the case of phobic dogs, no alterations in the gut microbiota were found, except for an increase in *Lactobacillus*, a bacterium that influences the production of the inhibitory neurotransmitter GABA [20]. The researchers suggested that these bacterial changes could lead to altered production of neuroactive metabolites, affecting behavior through interactions with the central nervous system. It is important to highlight that Mondo et al.’s study only demonstrated a correlation between altered gut microbiota composition and aggressive behavior but not a causal relationship between altered gut microbiota and the development of aggressivity in those dogs.

In humans, data demonstrating the influence of gut microbiota’s modulation on mental health and cognition remain scarce. For this purpose, Appleton [21] recently gathered some clinical studies which suggested the benefit of some probiotics on mood and mental health, reducing, for example, anxiety and depression scores. Probiotics are compounds which modify gut microbiota composition, and this issue will be discussed further in this review article.

In summary, the microbiota–gut–brain axis is a complex and dynamic system that facilitates communication between the gut and the brain, and its regulation can be beneficial for the treatment of gut or neurological disorders both in dogs and in humans.

#### 2.3.1. The Different Pathways in Microbiota–Gut–Brain Communication

There are several pathways involved in communication through the microbiota–gut–brain axis, metabolic, neurological, endocrine, and immunological pathways, as described below.

##### Metabolic Pathway

As previously mentioned, the gut microbiota and the host share a complex, mutually beneficial relationship. Gut bacteria break down undigested dietary fibers reaching the colon to produce metabolites like SCFAs, including acetate, propionate, and butyrate [8]. These SCFAs serve as primary energy sources for colon cells supporting intestinal balance and barrier function. Butyrate, for example, demonstrated to reinforce gut barrier, in vitro, by increasing tight junction protein assembly [22]. Furthermore, SCFAs may cross the blood–brain barrier (BBB), and they may impact the brain directly through several mechanisms, such as enhancing the BBB, regulating neurotransmission, and modulating the maturation and function of microglia [23]. By controlling gut inflammation and neuroinflammation, SCFAs may also help to mitigate neuronal hyperexcitability and thereby potentially reduce the frequency or severity of epileptic seizures [24].

##### Neural Pathway

The gut microbiota plays a key role in neural function by regulating neurotransmitter levels, including serotonin, dopamine, norepinephrine, and GABA [25]. Despite it being little plausible that the neurotransmitters produced in the gut may cross the BBB and act directly on the central nervous system (CNS), it is thought that the gut microbiota may indirectly influence the CNS and consequently some of its processes, including mood and behavior [25,26]. It seems reasonable that neural communication is possible thanks to the vague nerve or to the enteric nervous system [27].

GABA, an inhibitory neurotransmitter in both the central and enteric nervous systems, is produced by various lactic acid bacteria, including the *Lactobacillus*, *Enterococcus*, *Leuconostoc*, *Pediococcus*, *Propionibacterium*, and *Weissella* genera [20]. GABAergic neurotransmission helps to inhibit the amygdala, preventing inappropriate emotional and behavioral responses, and an imbalance in GABA regulation is associated with stress and anxiety [28]. This raises the following question: could GABA produced by lactic acid bacteria in the gut reach the amygdala (after crossing the BBB) or act through neurons projecting to the amygdala? In this context, to understand how GABA produced by lactic acid-producing bacteria could regulate emotional behavior, Blake et al. [19] carried out a study in mice, reporting that chronic treatment with the *Lactobacillus* strain decreased anxiety and depression behavior through the regulation of central GABA receptor expression in the amygdala and other regions of the brain but only in non-vagotomized mice. In addition to anxiety and depression, GABA imbalance has also been implicated directly in other brain disorders, including epilepsy [29], schizophrenia [30], and autism spectrum disorders [31].

Another very important neurotransmitter produced in the gut is serotonin. Over 90% of total human body serotonin is produced in the gut; consequently, only less than 10% is produced in the brain [32]. *Candida*, *Streptococcus*, *Escheridia*, and *Enterococcus* spp. can produce serotonin in the gut [32]. Serotonin is vital to functions like eating, sleep, cognition, social interactions, anxiety, and mood regulation [33]. It has been suggested that serotonin produced in the gut may activate 5-HT3 receptors in the vagal afferent fibers [27].

##### Endocrine Pathway

The gut microbiota, beyond modulating the previously mentioned pathways, also plays a vital role in regulating the endocrine systems connected to the gut–brain axis, specifically through the modulation of the hypothalamic–pituitary–adrenal axis (HPA). This complex system is essential to maintaining the body’s endocrine balance baseline and regulates various physiological processes in response to stress [34].

During stressful situations, the hypothalamus releases corticotropin-releasing hormone (CRH). This hormone then stimulates the pituitary gland to release adrenocorticotropic hormone (ACTH) into the bloodstream. ACTH, in turn, triggers the adrenal glands to produce cortisol, the body’s primary stress hormone [34]. Cortisol influences several functions, including immune responses, metabolism, and brain function [35].

The balance/imbalance of the HPA axis can be influenced by the gut microbiota. Certain bacteria may stimulate excessive activity within this system, potentially altering behavioral responses to stress. Additionally, stressful events and the subsequent activation of the HPA axis can lead to changes in the composition of the gut microbiota [27,34]. These changes in gut microbiota composition can compromise the integrity of the intestinal barrier, leading to increased intestinal permeability, allowing endotoxins and pathogens to enter the bloodstream, and triggering an uncontrolled inflammatory response. Inflammatory mediators like cytokines can cross the BBB and further activate the HPA axis, creating a feedback loop that worsens stress, inflammation, and dysbiosis [27].

##### Immune Pathway

Gut bacteria influence the activity and function of immune cells residing in both the gut and the brain. The gut microbiota plays a crucial role in activating immune cells and interacting with the immune system through pattern recognition receptors (PRRs). These receptors detect microbial components, triggering immune responses that include cytokine production, such as TNF-alpha [36]. These mediators can cross the BBB, affecting the central nervous system (CNS) by activating microglia, the brain’s resident immune cells, and potentially causing neuroinflammation, which may impact neuronal health and behavior [37]. In fact, neuroinflammation underlies several neurological disorders, such as Parkinson’s disease [38], Alzheimer’s disease [39], and epilepsy [40].

In summary, the microbiota–gut–brain axis represents a complex network of bidirectional communication involving the nervous system, the immune system, and the gut microbiota. This close interaction plays a crucial role in regulating mental health, behavior, and overall, the homeostasis of the organism.

### 2.4. Impact of Canine Gut Microbiota Imbalance on Health and Disease

As previously discussed, gut microbiota plays an essential role in both physiological and immunological functions, but it is unclear how it directly affects the pathogenesis of a disease state.

In a healthy state, a harmonious interplay and mutual regulation between the host and microorganisms maintains a balanced bacterial ecosystem. This equilibrium ensures that the gastrointestinal tract remains healthy, a state known as eubiosis, preventing the overgrowth of potentially pathogenic bacteria. However, when this delicate balance is disrupted, it leads to dysbiosis, as already mentioned before. This can result in alterations in bacterial metabolic activities and shifts in bacterial distribution within the gut [41].

Dysbiosis is observed in various diseases, including inflammatory bowel disease (IBD), obesity, allergy, and diabetes in humans and in animal models. However, it remains uncertain whether dysbiosis is a cause or a consequence of some diseases [41].

Suchodolski et al. [42], using the 454-pyrosequencing of the 16SrRNA gene and qPCR assays in fecal samples, found that dogs suffering from acute hemorrhagic diarrhea have higher levels of *Sutterella* and *Clostridium perfringens* and lower levels of *Blautia* and *Ruminococcaceae*. Some of these decreased bacteria produce butyrate and other SCFAs, which are crucial to gut homeostasis, as previously discussed.

Studies in dogs and cats indicate that alterations in the gut microbiota and its function are associated not only with gastrointestinal diseases, such as IBD, but also with disorders affecting other organ systems. These include chronic kidney disease [43], heart disease [44], brain disorders [45], diabetes mellitus [46], and obesity [47].

Advances in understanding the gut microbiota and their functions will pave the way for new diagnostic and therapeutic strategies in the future.

## 3. Canine Idiopathic Epilepsy

### 3.1. Definition of Canine Idiopathic Epilepsy

Epilepsy stands out as one of the most prevalent chronic neurological conditions affecting companion animals and humans. This disease is characterized by episodic and excessive electrical activity within the neuronal network. This activity leads to hypersynchronous activity, culminating in epileptic seizures. Such seizures are defined by Fisher et al. [48] as the occurrence of “at least two unprovoked seizures that are separated by more than 24 h”.

In dogs, the epileptic seizure consists of three phases: a preictal phase (indicator of forthcoming seizures), the ictus (seizure activity), and the postictal phase (where normal brain function is restored). During the postictal phase, the brain gradually returns to normal function. This phase can be brief or extended over several hours to days. Typically, the animal may appear disoriented, exhibit behavioral abnormalities such as repetitive vocalization or compulsive movement that fails to avoid obstacles, and show signs of fatigue, ataxia, hunger, or thirst. The animal may also express a need to urinate or defecate, seem exhausted, and sleep for an extended period. Postictal blindness or aggression may also occur [49].

Clinically, epileptic seizures often manifest with motor symptoms, including facial twitches, repeated jerking head movements and rhythmic blinking. These symptoms are frequently accompanied by heightened autonomic activity, such as dilated pupils, hypersalivation or vomiting, and behavioral alterations, for example, abnormal attention seeking from the owner. These clinical signs provide valuable insights for veterinarians for diagnosing and managing this condition in affected companion animals [49].

Recognizing the need for clarity and uniformity in defining and classifying this condition, the International Veterinary Epilepsy Task Force (IVETF) has taken proactive measures. They have formulated standardized definition, classification, and terminology through a consensus report, which aims to streamline the understanding of and approaches to epilepsy in veterinary medicine. This consensus report, as detailed by Berendt et al. [49], provides valuable insights into the diverse nature of epilepsy and underscores the importance of a unified framework for its diagnosis and treatment.

The IVETF has conducted a review of scientific research on idiopathic epilepsy, particularly focusing on cases with a genetic or suspected genetic basis [1].

Idiopathic epilepsy is the most common type of epilepsy in dogs, and the diagnosis of idiopathic epilepsy relies on factors such as the animal age at the time of seizure onset, unremarkable interictal and clinical examinations, and neurological evaluations, along with the diagnosis exclusion of other encephalopathies [49,50].

### 3.2. Composition of Gut Microbiota in Epileptic Dogs

The structure and composition of gut microbiota in epileptic dogs remain underexplored, but there is already some evidence that suggests a different gut microbiota composition on epileptic dogs when compared with healthy dogs, as presented in Table 1.

It is important to stress that many of the studies carried out so far aimed to assess how canine gut microbiota composition is influenced by some drugs, like phenobarbital [51], but they do not establish the comparison between the gut microbiota from epileptic dogs and the gut microbiota from healthy dogs (epilepsy-free dogs).

**Table 1:** Gut microbiota composition in a canine epilepsy context.

Author and Year	Results	Samples	Methods	References
Muñana et al.; 2020	No difference in *Lactobaccillus* species in stools collected from drug-naïve epileptic dogs vs. healthy dogs.	Fecal	16S rRNA gene amplicon sequencing	[52]
Garcia-Belenguer et al.; 2021	No difference in *Lactobaccillus* species in stools collected from epileptic dogs vs. healthy dogs.Reduced abundance of *Pseudomonadales*, *Prevotellaceae*, *Ruminococcaceae*, and *Peptococcaceae* in epileptic dogs vs. healthy dogs.	Fecal	16S rRNA gene amplicon sequencing	[5]
Garcia-Belenguer et al.; 2023	Higher abundance of *Lactobaccillus* genus in epileptic dogs vs. healthy dogs.	Fecal	16S rRNA gene amplicon sequencing	[53]

In a pilot study by Muñana et al. [52], it was reported that the abundance of fecal *Lactobaccillus* was not significantly different between epileptic dogs and healthy dogs.

Accordingly, Garcia-Belenguer et al. [5] did not report any substantial differences in the abundance of fecal *Lactobaccillus* between epileptic dogs and healthy dogs. However, the authors verified that beta diversity showed variations between healthy dogs and epileptic dogs after being treated with antiepileptics drugs or without treatment. These differences were not observed in the predominant bacteria but rather in those present in smaller quantities. The study revealed a decrease in the abundance of *Pseudomonadales*, *Prevotellaceae*, *Ruminococcaceae*, and *Peptococcaceae* in epileptic dogs when compared with control dogs. These differences are of particular interest because *Pseudomonas* bacteria produce GABA from glutamate [54]. Although GABA cannot cross the BBB, it may exert an indirect effect on the central nervous system through the vagal pathway [19], as explained before in the Neural Pathway Section. Thus, Garcia-Belenguer et al. stated that a decrease in GABA-producing bacteria may interfere with seizure frequency or severity. Lastly, *Peptococcaceae* and *Ruminococcaceae* are bacteria that produce SCFAs. SCFAs can cross the BBB, where they can regulate levels of GABA, glutamate, and glutamine, and play other important functions that may impact brain homeostasis [23], as previously explained in the Metabolic Pathway Section. These observations suggest a potential role of the gut microbiota in modulating neurotransmitter levels and in the pathophysiology of neurological disorders, such as idiopathic epilepsy.

In Jeffery et al.’s study [45], a decrease in *Prevotellaceae* was noted in dogs with meningoencephalomyelitis compared with healthy dogs. This finding aligns with Garcia-Belenguer’s study [5], which revealed a decrease in *Prevotellaceae* in epileptic dogs compared with healthy dogs. Therefore, this bacterial family could play a role in the prevalence of neurological diseases.

In a more recent paper by Garcia-Belenguer et al. [53], conflicting results were reported, since epileptic dogs showed higher abundance of *Lactobaccillus* genus compared with healthy dogs. This difference was attenuated after a ketogenic diet (a specific diet with promising results in idiopathic epilepsy, which will be discussed further in this review), over the course of a month.

## 4. Non-Conventional Interventions for Idiopathic Epilepsy in Dogs via Microbiota–Gut–Brain Axis Modulation

Since gut microbiota may be implicated in idiopathic epilepsy, its manipulation could potentially alter the disease’s progression.

While numerous antiepileptic drugs have been developed, approximately one-third of epilepsy cases in dogs are classified as drug-resistant [55]. This designation implies that seizure management remains ineffective even after the administration of two or more appropriate antiepileptic medications [55]. Moreover, many antiepileptic drugs promote adverse effects, such as sedation, ataxia, and polyphagia [56]. Consequently, there is an urgent demand for novel treatments that can ameliorate epileptic clinical signs in dogs.

Emerging research suggests that non-conventional interventions like the ketogenic diet, antibiotics, probiotics, and fecal microbiota transplant might offer new avenues for managing idiopathic epilepsy via the regulation of the microbiota–gut–brain axis.

Subsequently, some studies have investigated the impact of gut microbiota-targeted therapeutical interventions for epilepsy in dogs, namely, for seizure control, as described in Table 2 and discussed below.

**Table 2:** Studies on gut microbiota-targeted therapeutical interventions for idiopathic epilepsy in dogs.

Author and Year	Strategy Used		Type of Study	Results	References
Garcia-Belenguer S. et al., 2023	Ketogenic diet	Medium-chain triglyceride (MCT)-enriched diet	Pilot, non-blinded, no-placebo, prospective study in two groups (epileptic vs. non-epileptic dogs)	MCT diet promoted a modification of gut microbiota composition in epileptic dogs	[53]
Berk et al., 2020	Ketogenic diet	Medium-chain triglyceride (MCT)-enriched diet	Multicenter, prospective, randomized, double-blinded, placebo-controlled study	Seizure frequency lower in dogs fed MCT diet than in dogs fed control diet	[57]
Molina et al., 2020	Ketogenic diet	MCT-enriched diet	Prospective, open-label, single-arm study with no placebo	Seizure frequency lower in dogs fed MCT diet as an adjunct to antiseizure drugs (ASDs)	[58]
Law et al., 2015	Ketogenic diet	MCT-enriched diet	Prospective, randomized, double-blinded, placebo-controlled study	Seizure frequency lower in dogs fed MCT diet than in dogs fed placebo	[59]
Nakatsuka et al., 2023	Ketogenic diet	Medium-chain triglyceride (MCT)-enriched diet	Prospective, randomized, double-blinded, placebo-controlled study	Data not statically significant—only a slight reduction in seizure frequency and duration in dogs on MCT diet concurrent with zoniazide (an antiepileptic drug)	[60]
Packer et al., 2016	Ketogenic diet	Medium-chain triglyceride (MCT)-enriched diet	Prospective, randomized, double-blinded, placebo-controlled study	Reduction in some behavioral comorbidities related to epilepsy (which resemble attention-deficit /hyperactivity disorder, ADHD, in humans and rodent models) in dogs fed MCD diet	[61]
Schmidt et al., 2023	Probiotics	*Bifdobacterium longum*	Prospective, randomized, double-blinded, placebo-controlled study	This paper reports on a study design: “The datasets and data analysis will be published separately”	[62]
Ledeganck et al., 2022	Antibiotics	Amoxicillin–clavulanic acid	Preliminary study (very few animals enrolled in the study without control group)	Seizure frequency was reduced in epileptic dogs	[63]
Watanangura et al., 2024	Fecal microbiota transplantation (FMT)		Open-label, prospective, pilot study	FMT alleviated behavioral comorbidities related to epilepsy, such as ADHD, as well as fear and anxiety-like behavior in drug-resistant epileptic dogs	[64]

### 4.1. Ketogenic Diet

Studies conducted in animal models have shown that epileptic animals often exhibit abnormalities in glucose utilization and metabolism within the brain and that these metabolic impairments may lead to a reduction in energy production and to increased susceptibility to seizures [65]. Although a causal link has not been fully established yet, Samokhina et al. [66] reported that chronic hypometabolism can promote epileptogenesis in mice. It is thought that metabolic impairments in “epileptic brains” may be varied, for example, glycolysis impairment and decreased pyruvate dehydrogenase activity [65]. Since the brain is an organ metabolically expensive and, as far as it is known, it uses a considerable amount of energy for electrical signaling [67], those metabolic problems may compromise the stabilization of membrane potentials and the regulation of neural signaling [65]. It is worth noting that the brain energy requirements differ among species and, for example, the cost of learning is not the same across species [67].

Ketogenic diets, used to manage epilepsy, are high in fat but low in protein and carbohydrates. This keeps blood glucose levels low, prompting the liver to produce ketone bodies like β-hydroxybutyric acid from fatty and amino acids; in other words, ketosis is promoted [68]. These ketone bodies can be used as auxiliary brain fuel in addition to glucose. Adding medium-chain triglycerides (MCTs) to a regular diet with carbohydrates offers a new way to provide additional brain fuel. MCTs are quickly hydrolyzed in the gastrointestinal tract and the resulting medium-chain fatty acids (MCFAs) are rapidly absorbed into the portal vein, reach the liver and extrahepatic tissues, and are converted to ketone bodies by the liver. Unlike long-chain fatty acids, which are metabolized more slowly, MCFAs can enter the TCA cycle without needing pyruvate dehydrogenase activity, like ketone bodies. This provides a stable and efficient energy source for the brain. Therefore, a ketogenic diet can meet the brain’s energy needs in a stable way, being beneficial as an antiseizure strategy [68]. These findings suggest that MCTs could play a key role in managing canine epilepsy, potentially improving quality of life for these animals [68].

The use and potential benefit of MCTs in dogs with idiopathic epilepsy has been explored in some studies [57,58,59]. In these studies, MCT diet revealed to be quite successful, since 9 to 14% of dogs achieved freedom from seizure and 13 to 43% experienced at least a 50% reduction in seizure frequency [57,58,59]. The variety of basal diets used in these studies makes it difficult to draw definitive conclusions.

The recent study of Nakatsuka et al. [60] aimed to evaluate the impact of feeding dogs which presented a drug-resistant idiopathic epilepsy and were treated primarily with zonisamide (a well-known antiepileptic drug) a commercially available therapeutic MCT-rich diet (Purina Pro plan NC Neurocare, Nestlé Purina, USA). Despite the fact that the study did not find any statistically significant difference in epileptic seizure frequency or duration between the standard placebo diet and the Neurocare diet, the analysis of individual data hinted that feeding zonisamide-treated dogs a Neurocare diet may lead to a reduction in seizure frequency and duration, particularly in the final thirty days of the three-month trial period. Furthermore, there was a noticeable increase in serum β-hydroxybutyric acid levels at the conclusion of the Neurocare diet phase compared with the placebo diet phase. β-Hydroxybutyric acid might provide an alternative energy source and contribute to seizure reduction [68]. Despite it being suggested that the Neurocare diet is a safety treatment option for dogs concomitantly with antiseizure medication in resistant idiopathic epilepsy, it is important to note that the limited number of animals enrolled in this study makes this study not very solid.

From a little different perspective, a study conducted by Packer et al. [61] aimed to evaluate whether the ketogenic diet could be advantageous to reduce behavioral comorbidities of epilepsy in dogs that resemble attention-deficit/hyperactivity disorder (ADHD) in humans and rodent models. This study suggested that the MCT diet was successful in reducing some behavioral symptoms, such as chasing and stranger-directed fear in epileptic dogs.

Although the ketogenic diet has shown a favorable effect on epilepsy symptom control, the mechanisms behind its effect remain poorly clarified.

It has been suggested that the antiseizure effect of the ketogenic diet can also rely on the regulation of gut microbiota composition. In this context, a study conducted by Xie et al. [69] suggested that the ketogenic diet can be beneficial for epileptic children through the reshaping of the gut microbiota. Accordingly, Olson et al. [70] demonstrated that the ketogenic diet alters the gut microbiota in epileptic mice. This diet modifies the gut microbiota composition, and this alteration promotes the increase in the levels of GABA and glutamate, which, in turn, provides protection against seizures. Additionally, Pilla et al. [71] showed that dogs on the ketogenic diet presented an increase in *Bacteroidaceae*, compared with dogs on a standard diet. Garcia-Belenguer S. et al. [53] suggested that the consumption of an MCT diet significantly reduces the abundance of the *Actinobacteria* phylum in epileptic and non-epileptic dogs, which may contribute to seizure reduction. Moreover, higher relative abundance of the *Negativicutes* class and the *Selenomonadales* order in epileptic dogs compared with non-epileptic dogs after the MCT diet was also observed. The authors suggested that those taxa can be used as biomarkers related to the MCT diet in the future. We must be careful, as the studies mentioned above (Pilla et al. and Garcia-Belenguer S. et al.) just showed an expected correlation between gut microbiota modification and seizure reduction and not a causal relationship between these factors.

Therefore, while the ketogenic diet seems to be an advantageous adjuvant strategy for the treatment of idiopathic epileptic dogs, there is a need to further research the mechanisms behind its effect and the adverse effects related to long-term use, such as kidney stones and loss of bone mineral content [72].

#### 4.1.1. Probiotics

Probiotics, composed of beneficial microorganisms for intestinal health, mainly consisting of *Lactobacillus*, *Bifidobacterium*, and *Sacharomycetes*, have attracted increasing interest in the field of veterinary neurology, particularly in cases of canine idiopathic epilepsy [73].

Recent studies started to explore the link between probiotics and seizure management in dogs, offering a new hope for the owners of pets facing this condition [62].

A promising study by Bagheri et al. [74] conducted in epileptic rats revealed a significant reduction in seizure severity and an attenuation of cognitive impairment in animals receiving a probiotic supplement. These findings suggest that probiotics could be beneficial in the context of epilepsy. The precise mechanisms by which probiotics exert their beneficial effects are not yet fully understood, but several hypotheses have been proposed. It is possible that probiotics act by strengthening the intestinal barrier, reducing systemic inflammation, or producing beneficial metabolites for the brain.

A very recent study by Sharkawy et al. [75] revealed that probiotics reduced the seizure frequency, reduced seizure severity and improved the quality of life of children with drug-resistant epilepsy.

However, despite these encouraging results obtained in animal models and in clinical trials with humans, it is important to note that to date, there are no published results reporting the benefit of probiotics on the attenuation of seizures in dogs, so additional research is urgently needed to determine the effects of probiotics on the health and well-being of epileptic dogs.

Furthermore, not all probiotics have the same composition, and their efficacy may vary depending on the strain used, dosage, and the duration of treatment.

In conclusion, while probiotics offer new hope in the management of canine health, their use must be cautious and based on solid scientific evidence. With further research and development, probiotics could potentially become a valuable tool in the therapeutic arsenal to help dogs suffering from epilepsy to have healthier and happier lives.

#### 4.1.2. Antibiotics

A study by Ledeganck et al. [63] investigated the effects of antibiotics on seizure frequency in dogs with antiseizure-drug resistance. This study arose when a dog diagnosed with idiopathic epilepsy resistant to anticonvulsant medication presented at a veterinary hospital for suspected infection. The dog was prescribed antibiotics (amoxicillin–clavulanic acid) for a month. During the treatment, the dog did not experience any epileptic events, despite having a seizure frequency of 2.5 per week before the treatment. Upon suspension of the antibiotic treatment, seizures re-occurred. Subsequently, four other dogs with antiseizure-drug resistance were treated with antibiotics (amoxicillin–clavulanic acid) for 33 days. Surprisingly, the researchers observed a reduction in seizures following antibiotic treatment. Two dogs experienced a complete cessation of seizures, one showed an 80% reduction in seizure frequency, while the last one experienced an increase in seizure frequency. The latter dog underwent a diet change during treatment, which could explain the outcome, given that as discussed before, a specific diet may influence gut microbiota composition and the modification of gut microbiota composition may have impact on the brain, in particular in an epilepsy context, as suggested in a recent pilot study in drug-naïve children [76]. Unfortunately, the mentioned study conducted by Ledeganck et al. [63] lacked fecal analysis, which could have provided insights into the composition of the gut microbiota before and after antibiotic treatment, thus allowing a better understanding of its influence on epilepsy. Furthermore, this study enrolled only a few animals (four dogs), did not show any control group, and had many confounding factors, so the conclusions drawn have no statistical support, corresponding only to anecdotical observations.

Until now, data revealing the positive effect of antibiotics on epileptic dogs are scarce. Thus, it is imperative to further elucidate the long-term effects of antibiotic therapy in epileptic dogs. Furthermore, it is crucial to select the appropriate antibiotics, since certain antibiotics, like cephalosporins and beta-lactams, can induce seizures due to their direct or indirect inhibitory effects on GABA.

On the other hand, the use of antibiotics, particularly long-spectrum ones, may promote side effects, such as antibiotic resistance, which constitutes a public health problem, and this cannot be neglected.

#### 4.1.3. Fecal Microbiota Transplant

Fecal microbiota transplant corresponds to the transfer of feces from a healthy donor into a patient with a diseased gastrointestinal tract to restore the gut microbiota composition of the patient and consequently ameliorate the symptoms of a specific gut condition. This strategy is already used in humans and has given proof of efficacy in many patients [77]. Some authors consider that this strategy can be applied successfully in epileptic patients, although evidence is scarce. One study conducted by He et al. [78] demonstrated for the first time that a patient with Crohn’s disease (a severe type of inflammatory bowel syndrome) who also suffered from epilepsy became seizure-free after fecal microbiota transplant. Obviously, these data report only one case of a single patient, and it will be necessary to confirm these results with more robust studies.

Very recently, Watanangura et al. [64] conducted for the first time a pilot study to evaluate the impact of fecal microbiota transplant on behavioral comorbidities, such as fear and anxiety-like behavior, in drug-resistant epileptic dogs. In this study, fecal microbiota transplant demonstrated to be efficient in reducing the behavioral comorbidities of epileptic dogs, reducing, for example, their impulsivity and increasing the quality of life of dogs and their owners. It is important to highlight that the feces donors used in this study were from dogs with idiopathic epilepsy which had been treated only with phenobarbital and had been seizure-free for more than a year. After fecal microbiota transplantation, behavioral improvement was accompanied by a modification of gut microbiota composition and of some neurotransmitter levels, like GABA and glutamate. However, the frequency and severity of seizures were only improved in some dogs. It is important to note that this study enrolled a very small number of animals; therefore, the results are devoid of relevance from a statistical point of view.

These studies showed that fecal microbiota transplant can be envisaged as a promising approach to the treatment of epilepsy in humans and in dogs, but double-blinded, randomized controlled studies are still needed in the future.

## 5. Future Perspectives and Conclusions

The emerging research on the role of the gut microbiota in canine idiopathic epilepsy underscores the intricate relationship between gut health and neurological conditions. Evidence suggests that the dysregulation of the gut microbiota may contribute to the pathogenesis of epilepsy in dogs and that the modulation of gut microbiota composition, through interventions such as dietary changes, probiotics, antibiotics, or fecal microbiota transplant, could offer promising avenues for seizure management.

While studies have shown encouraging results in terms of reducing seizure frequencies through microbiota modulation, particularly in animal laboratory models and in humans, further research is needed to elucidate the underlying mechanisms and long-term effects of these interventions in dogs. Fecal analysis before and after treatment, along with comprehensive clinical studies, could provide valuable insights into the dynamics of the gut–brain axis and its influence on epilepsy. Moreover, it is essential to consider potential side effects and drug interactions when implementing microbiota-modulating therapies in epileptic dogs. The careful selection of antibiotics, specific diet, or probiotics is imperative to ensure the safety and efficacy of these interventions.

Overall, the exploration of the gut microbiota’s impact on canine idiopathic epilepsy represents a promising frontier in veterinary medicine. By deepening our knowledge on this complex interplay, we may uncover novel therapeutic strategies that improve seizure control and enhance quality of life for dogs living with epilepsy.
